# The Growth Response of Two Diatom Species to Atmospheric Dust from the Last Glacial Maximum

**DOI:** 10.1371/journal.pone.0158553

**Published:** 2016-07-06

**Authors:** Tim M. Conway, Linn J. Hoffmann, Eike Breitbarth, Robert F. Strzepek, Eric W. Wolff

**Affiliations:** 1 British Antarctic Survey, Cambridge, United Kingdom; 2 Department of Earth Sciences, University of Cambridge, Cambridge, United Kingdom; 3 Department of Earth Sciences, ETH Zürich, Zürich, Switzerland; 4 Department of Chemistry, University of Otago, Dunedin, New Zealand; Laval University, CANADA

## Abstract

Relief of iron (Fe) limitation in the surface Southern Ocean has been suggested as one driver of the regular glacial-interglacial cycles in atmospheric carbon dioxide (CO_2_). The proposed cause is enhanced deposition of Fe-bearing atmospheric dust to the oceans during glacial intervals, with consequent effects on export production and the carbon cycle. However, understanding the role of enhanced atmospheric Fe supply in biogeochemical cycles is limited by knowledge of the fluxes and ‘bioavailability’ of atmospheric Fe during glacial intervals. Here, we assess the effect of Fe fertilization by dust, dry-extracted from the Last Glacial Maximum portion of the EPICA Dome C Antarctic ice core, on the Antarctic diatom species *Eucampia antarctica* and *Proboscia inermis*. Both species showed strong but differing reactions to dust addition. *E*. *antarctica* increased cell number (3880 vs. 786 cells mL^-1^), chlorophyll *a* (51 vs. 3.9 μg mL^-1^) and particulate organic carbon (POC; 1.68 vs. 0.28 μg mL^-1^) production in response to dust compared to controls. *P*. *inermis* did not increase cell number in response to dust, but chlorophyll *a* and POC per cell both strongly increased compared to controls (39 vs. 15 and 2.13 vs. 0.95 ng cell^-1^ respectively). The net result of both responses was a greater production of POC and chlorophyll *a*, as well as decreased Si:C and Si:N incorporation ratios within cells. However, *E*, *antarctica* decreased silicate uptake for the same nitrate and carbon uptake, while *P*. *inermis* increased carbon and nitrate uptake for the same silicate uptake. This suggests that nutrient utilization changes in response to Fe addition could be driven by different underlying mechanisms between different diatom species. Enhanced supply of atmospheric dust to the surface ocean during glacial intervals could therefore have driven nutrient-utilization changes which could permit greater carbon fixation for lower silica utilization. Additionally, both species responded more strongly to lower amounts of direct Fe chloride addition than they did to dust, suggesting that not all the Fe released from dust was in a bioavailable form available for uptake by diatoms.

## Introduction

Over at least the last 800,000 years, glacial-interglacial cycles in earth climate have been coincident with and largely driven by regular 80–100 ppmv changes in the levels of atmospheric CO_2_ [1–3]. Understanding how atmospheric CO_2_ changes during glacial inception or termination has thus been a focus of much research, with a number of hypotheses put forward to explain the cycling [4,5]. One such idea, the ‘iron hypothesis’ suggested that enhanced dust flux to the oceans during glacial intervals could have acted to relieve Fe-limitation of phytoplankton in some areas of the surface oceans, thereby increasing nutrient utilization and carbon export [6,7]. This increased carbon storage in the deep ocean could in return have lowered atmospheric CO_2_ over glacial timescales [6]. This hypothesis centered on three High Nutrient Low Chlorophyll (HNLC) regions of the surface ocean where today vanishingly-low dissolved Fe concentrations limit growth, while upwelling ensures macronutrients are found in excess in surface waters but does not supply sufficient dissolved Fe to utilize these macronutrients [8]. Although this hypothesis has been somewhat superseded by later ideas which instead suggest a greater role for upwelling and circulation control on deep ocean carbon storage during glacial intervals [9–11], the most recent studies suggests that Fe-fertilization may still play an important role in moderating atmospheric CO_2_ on glacial and millennial timescales [12–16].

The Southern Ocean is the largest of the HNLC regions, and Antarctic and sub-Antarctic surface ocean waters today often experience very low dissolved Fe concentrations (0.05–0.2 nmol L^-1^; [17–19]), in large part due to the very low modern annual input of desert dust to Southern Ocean waters (e.g. modelled to be <0.2 g m^2^ yr^-1^; [20]). Flux of atmospheric dust to Antarctica was up to 50 times greater during glacial intervals during the past 800,000 years [7,21,22], and the flux to the nearby Southern Ocean was also significantly enhanced during glacial intervals over at least the last four million years [14]. Ice core records demonstrate that greater dust fluxes would have meant significantly greater delivery of total Fe and seawater-soluble Fe to Antarctica and therefore to nearby surface Southern Ocean waters [23–25]. As the Southern Ocean is one of the largest sinks of atmospheric CO_2_ [26], understanding the effect of natural Fe sources on primary production of important primary producers can help us to understand the role of the Southern Ocean in the global carbon cycle in the past, today, and in the future.

Several *in situ* Fe fertilization experiments in all HNLC regions have proven that once Fe limitation is relieved, total phytoplankton primary production, biomass, and photosynthetic efficiency increase rapidly [27]. However, individual species react differently to Fe fertilization and not all of them seem to benefit in the same way so that species composition can change significantly. Generally, large diatom species benefit most and quickly dominate the phytoplankton communities in all HNLC regions after Fe fertilization, despite the large physicochemical, geological, and biological differences between the Subarctic Pacific, the equatorial Pacific, and the Southern Ocean [28–30]. However, small diatom species also increased growth during the Southern Ocean Fe fertilization experiment EIFEX [31]. More recent investigation of the effect of natural Fe fertilization events such as volcanic eruptions, island sediment inputs, seabird guano, and desert dust storms have shown that care must be taken when directly extrapolating the effects of artificial Fe fertilization experiments to natural events [32–35]. The biological effect of a certain amount of Fe added to a natural system can be very different, as the bioavailability of Fe depends on not only Fe concentration, but also seawater pH, the organic ligand concentration in the water, and the chemical form the Fe is delivered in [36].

The complexities of assessing concentration, solubility, and eventually bioavailability of Fe from dust, and then applying these parameters through experiments using additions of simply dissolved Fe, can obscure the simplicity of the question we would like to answer, that is: do additions to seawater of dust of the sort that was added to the Southern Ocean in glacial periods actually enhance productivity? The aim of the present study was therefore to investigate how different Antarctic diatom species would respond to the addition of natural atmospheric dust from a glacial interval. In order to achieve this, we extracted Antarctic dust from the Last Glacial Maximum of the EPICA Dome C ice core using novel sublimation techniques, and added this dust to cultures of the Antarctic diatoms *Eucampia antarctica* and *Proboscia inermis*. We compared the growth response of both species with those in control and FeCl_3_-amended treatments. Incubations were carried out under representative light and temperature conditions in natural Sub-Antarctic seawater without the use of complexing ligands such as ethylenediaminetetraacetic acid (EDTA) or siderophore desferrioxamine B (DFB), which are typically used to complex metals in culture experiments [37–39].

## Materials and Methods

Ice-sampling and dust extraction was carried out at the British Antarctic Survey and Department of Earth Sciences, University of Cambridge [24]. Diatom incubations and sampling were carried out at the Department of Chemistry, University of Otago.

### EPICA Dome C Dust Samples

#### Ice and Dust Samples

Antarctic dust of Last Glacial Maximum age (LGM) was obtained from the European Project for Ice Coring In Antarctica (EPICA) Dome C ice core from Dome C on the East Antarctica Plateau (EDC; 75°06’S 123°21’E, 3,233 m asl). This ice core provides a climate and dust archive stretching back 800,000 years [1–3,21,25]. For this study, we chose ice samples to cover the highest-dust portion of the LGM (21–26 kyr BP; [21]), with dust concentrations of 0.6–1 mg kg^-1^ [21], providing sufficient Fe to observe any possible response in culture. Despite these relatively-large dust concentrations compared to other intervals [21], it was still necessary to combine dust from several kg of ice to provide enough dust for incubations. Ice was supplied by EPICA as 8 bags (55 cm length) of ‘A cuts’ (approx. ¼ of the core), with ages of 22.1–22.3 and 24.7–25.8 kyr BP [40]. We calculated that the total mass of dust provided by the ice was 2.84 mg, based on the high-resolution dust record of Lambert et al. [21]. Sub-samples of ice from these bags and ice of similar age were sampled and analysed for total and soluble Fe concentrations in our recent study on Fe solubility [24], allowing us to calculate the soluble Fe that would be expected to be released in seawater. There is large variability in both total and soluble Fe at high-resolution in the ice core, which means it is difficult to calculate a representative average soluble Fe per 55 cm bag accurately (e.g. the Fe which dissolves at pH 5 ranges from 1.5–28 ng g^-1^ for these EDC bags (mean 9±9 ng g^-1^; 1SD)) [24]. However, based on measured Fe values and the weight of ice used from each bag, we estimate that the total mass of dust available from these EDC bags would provide ~0.2 mg of total Fe and ~0.02 mg of soluble Fe available to be used for culture experiments [24].

#### Ice Sample Processing and Laboratory Procedures

EDC ice samples were cut with a band saw and then decontaminated within a class 100 flow hood in a cold room (-20°C) at the British Antarctic Survey according to published methods [24]. All plastic used was rigorously acid cleaned in the Department of Earth Sciences, University of Cambridge, and strict handling procedures were used to minimize contamination [24]. Briefly, each piece of ice was handled with acid-cleaned polyethylene gloves and scraped sequentially with ceramic blades to remove the outer edges of ice, which are contaminated with trace metals by collection, drilling and handling [24,41]. Ice samples were then taken for sublimation [24], which allows extraction of the dust without melting of the ice. This is important because release of Fe from dust is strongly pH dependent, and Fe solubility and speciation can be affected by exposure of dust to water [24,42]. Thus dry-extraction, together with the fact that the major deposition mode of dust deposited at Dome C during the LGM was dry [25], means that the dust used in this study should be representative of natural aerosol dust that would have been deposited to the nearby Southern Ocean at the LGM.

Decontaminated ice samples were sublimated using the larger ice mass configuration described by Conway *et al*. [24] (see S1 Fig). Sublimation of ice was achieved by lowering the pressure below the triple-point of water (0.01°C, 6.1173 mbar). Briefly, decontaminated ice samples were placed within open 1 L wide-mouthed Nalgene low-density polyethylene (LDPE) bottles. The 1 L bottles were placed within closed 4 L high-density polyethylene bottles (with holes drilled in the lids). The 4 L bottles were placed within a Schott glass desiccator and the desiccator closed. All transfer of ice to the desiccator was carried out under class 100 filtered air at -20°C. The sealed desiccator was carried out to the warm lab and attached to a vacuum pump and freezer trap [24] (see S1 Fig). The pump was turned on, the pressure was reduced to 0.8–1.6 mbar and the ice was allowed to sublime, following procedures described previously. Once all the ice had gone, and the pressure had returned to the background of the chamber (0.27 mbar), the desiccator was closed, the pressure released and the desiccator returned to the flow bench in the cold room for opening and addition of more ice. Sublimation proceeded at a rate of ~ 10 g hr^-1^, meaning that it required several weeks to extract enough dust for incubations, with fresh ice added to the same bottle every ~2 days. Testing established that Fe contamination by sublimation was very low (<40 pg per ~500 g 2 day session [42]), which is insignificant compared to the micrograms of seawater-soluble Fe that are present from dust within 500 g of ice [24].

### Diatom Incubations

#### Laboratory Procedures

All incubation processing and sampling of incubation materials was carried out under class 100 laminar airflow in the Department of Chemistry at the University of Otago, using strict clean procedures to prevent trace metal or biological contamination. All plastic equipment and pipette tips in contact with incubations was rigorously acid-cleaned using standard methods [24,43,44], which included rinsing thoroughly with 1% v/v quartz-distilled hydrochloric acid, ultrapure water (UPW; 18.2 MΩ) and clean sub-Antarctic seawater (see below) prior to use.

#### Seawater

Natural sub-Antarctic seawater for incubation experiments was collected onboard the R/V Polaris II on the 14th July 2009 from a station at the distal end of the Munida Transect, close to New Zealand (45.8°S 171.0°E; [45]; for which permission was not required). Water was collected using trace-metal clean towfish and sampling lines [46], filtered with 0.45 μm Pall Aquaprep 600 capsules into acid-cleaned polycarbonate carboys (in the dark) onboard ship and frozen at -20°C to preserve organic ligands. Prior to use in incubations, the seawater was thawed and filtered with Sartobran 150 filter cartridges (0.2 μm with 0.45 μm prefilter), before being used in incubation experiments in July-August 2009. The dissolved Fe concentration in the seawater (low-Fe seawater) was 0.223 ± 0.023 nmol L^-1^, measured by CL-FIA (see later) and the organic ligand concentration was approx. 0.5 nmol L^-1^, measured by competitive ligand stripping voltammetry [47]. The pH of the seawater was 8.05, measured using photometric methods [48].

#### Diatom Species

For this study, we chose monospecific cultures of two medium-large diatom species *Eucampia antarctica* (50–100 μm) and *Proboscia inermis* (150–200 μm) which are both important members of the modern Antarctic diatom assemblage [11,49–52]. *E*. *antarctica* has a well-documented bloom response to Fe and was also a widely distributed species at the LGM [11,50,53,54]. In fact, *E*. *antarctica* abundance in sediment cores has been found to be several fold greater during glacial intervals, with *E*. *antarctica* comprising up to 40% of the diatom assemblage [11]. The record of *P*. *inermis* is less well documented, as is the species’ response to Fe addition; however, both a growth and lack of growth response to Fe have been described previously [39,55]. We chose these species in order to test the effect of Fe from atmospheric dust on diatoms which are known to have different Fe requirements in order to gain a more realistic picture of how the whole diatom assemblage at the LGM would respond to dust addition. The cultures used in this study were isolated from the Southern Ocean (57°51’S, 139°50’E) in 2001 [39] and had been grown at 3°C in Aquil media at the University of Otago. Each culture was grown into Fe limitation for a number of generations in sterile 0.22 μm filtered low-Fe seawater, with only major nutrients (nitrate, phosphate and silicate) added to the culture. Fe limitation in cultures prior to incubation experiments was confirmed by measurement of the quantum use photosynthetic efficiency of photosystem II (Fv/Fm) at values less than 0.3 [56,57].

#### Incubation Setup

Diatom culture incubations were designed to match natural conditions as closely as possible. We used natural sub-Antarctic seawater without EDTA to complex dissolved metals and used realistic constant light (30 μmol photons m^-2^ s^-1^ from cool fluorescent tubes) and temperature (3°C) conditions. In addition to incubations with added EDC dust, control incubations (with only macronutrient addition to represent Fe-limited conditions) and incubations with addition of dissolved Fe standard (FeCl_3_; to assess the bioavailability of dissolved Fe) were carried out under identical conditions.

Incubation media were prepared in six acid-cleaned 8 L polycarbonate canisters, three for each diatom species (control, +dust and +FeCl_3_ conditions). Each bottle was filled with 6 L of low-Fe seawater, and 650 μL of each of silicate, nitrate and phosphate solutions added to make final concentrations of ~40 μmol L^-1^ silicate and nitrate and ~3 μmol L^-1^ phosphate. The nutrient solutions were either cleaned prior to addition with Chelex ion-exchange resin to remove dissolved Fe (nitrate, phosphate) or were low-metal standard solutions (silicate). Following mixing, the canisters were amended with either 0.5 L of a seawater-dust suspension (+dust), 0.5 L of low-Fe seawater and an aliquot of FeCl_3_ solution (+FeCl_3_) or simply 0.5 L of low-Fe seawater (control). The seawater-dust suspension was prepared by addition of 1 L of low-Fe seawater to the 2.84 mg of extracted EDC dust (present in three 1 L LDPE bottles), with extensive shaking/sonication in the bottles. The seawater-suspension was then combined and subdivided so that 500 mL of the mixture was added to each of the two +dust canisters, corresponding to a calculated average dust addition of 0.22 mg L^-1^ dust. The measured dissolved Fe concentration by CL-FIA (see later) immediately after dust addition for the two +dust canisters was 14 and 9 nmol L^-1^. Addition of FeCl_3_ to the +FeCl_3_ canisters was designed to match these values, by addition of aliquots of a 179 μmol L^-1^ FeCl_3_ solution (prepared by 100x dilution of a 17.9 mmol L^-1^ FeCl_3_ 2% HNO_3_ solution with UPW) to each canister.

A 3.25 mL inoculum of *E*. *antarctica* or *P*. *inermis* culture was pipetted into each canister, and the contents gently mixed. *P*. *inermis* was added to the 9 nmol L^-1^ dissolved Fe +dust or +FeCl_3_ canisters, and *E*. *antarctica* to the 14 nmol L^-1^. Following mixing, the canisters were subdivided into five 1 L transparent polycarbonate bottles, providing 5 replicate bottles for each incubation, and the remaining volume in the canister taken for measurement of a number of initial parameters (see below). The 1 L incubation bottles were sealed, double bagged within transparent ziplock bags and placed in thermostat controlled circulating water baths. Bottles were gently mixed and re-arranged within the incubator once per day to ensure constant light. Incubations were designed to last for 18 days but were stopped at 12 (*E*. *antarctica*) and 17 (*P*. *inermis*) days to prevent nutrient limitation, based on visual observation of extensive diatom biomass.

#### Sub-sampling for Growth Parameters

Cell number, chlorophyll *a*, dissolved Fe concentration (dFe), macronutrient concentrations, and the quantum use efficiency of photosystem II (Fv/Fm) were measured throughout the experiment at regular intervals. For each treatment, these parameters were measured in subsamples from 3 of the 5 incubation bottles. Subsamples for cell number and Fv/Fm were taken approx. every 2 days, and for the other parameters approx. every 4 days. The initial canisters were sampled for each of these parameters and particulate organic carbon (POC) at the beginning of the experiments, and all 5 incubation bottles were subsampled for these parameters, together with cell surface area/volume and POC concentration at the completion of each incubation.

#### Growth Parameters and Analysis

Photosynthetic quantum-use efficiency of photosystem II (Fv/Fm) provides a sensitive indicator as to whether a culture is Fe-stressed, and responds rapidly to relief of Fe-limitation [58]. This parameter has been used in a range of recent studies to demonstrate the effect of ash-fertilisation on phytoplankton [59,60,32] and Fe-limitation in the open ocean [61], with values ≤0.3 considered to be indicative of Fe-limitation [59,61]. Fv/Fm in this study was measured using a PhytoPAM (Walz, Germany) following dark adaptation of samples at low temperature, after Kolbowski and Schreiber [62]. Unfortunately, Fv/Fm data is only available from later stages of the experiments due to analytical issues with the PhytoPAM. Diatom cell number, length and width were determined using light microscopy in small volumes of solution (5–20 mL) after fixation with acid Lugol’s solution [63]. Cell surface areas and volumes were then calculated following either Hillerbrand *et al*. [64] for *P*. *inermis*, or using the ideal shape calculations described in S1 Text for *E*. *antarctica*. In the latter case we chose to use this calculation to ensure inclusion of the large horns of *Eucampia* sp., which visibly contain cytoplasm material. The cultures were not axenic but were visibly checked by microscope both before and during incubations, and no bacterial growth or any other phytoplankton species were present at any point.

Subsamples for determination of chlorophyll *a* and macronutrient concentration were filtered using GF/F filtration, and both the filter and filtrate were frozen at -20°C for later analysis for chlorophyll *a* and macronutrients respectively. Macronutrient concentrations (nitrate, phosphate and silicate) were determined using standard photometric methods [65]. Prior to analysis of chlorophyll *a*, the frozen filters were placed in 10 mL of 90% acetone in polypropylene vials, vortexed and then re-frozen at -20°C for 18–24 hours. Samples were allowed to melt, centrifuged at -5°C for 3 minutes (4100 rpm), kept in the dark and the supernatant pipetted into a glass vial for chlorophyll *a* analysis by a Turner fluorometer using standard procedures [66]. Subsamples for POC were filtered through pre-combusted GF/F filters and the filter frozen at -85°C. Filters were then dried at 65°C and stored dry until analysis with standard methods [67]. A blank value for POC was obtained from unused pre-combusted GF/F filters, and this was subtracted from all measurements.

Subsamples for measurement of dissolved Fe were collected with acid-cleaned polypropylene syringes, and filtered through 0.2 μm Millex GP PES Express Sterile 3 mm filtration units, which were rinsed with quartz-distilled HNO_3_ and UPW prior to use. Samples were collected into 125 mL LDPE bottles, acidified to pH ~1.6 with quartz-distilled HCl and were stored for several months before analysis. Dissolved Fe concentrations were determined in acidified seawater using chemi-luminescence flow injection analysis (CL-FIA) with luminol (5-amino-2,3-dihydro-1,4-phthalazine-dione) modified by Steve Rusak (pers. comm.) from previous studies [68,69]. Samples were pre-concentrated onto a Toyopearl AF-chelate resin column, analysed with a Waterville Analytical flow injection analyser and calibrated using standard additions, prepared daily. The limit of detection was defined as three times the standard deviation of the mean of a series of replicate analysis on a given sample. The method was checked by measurement of the NASS-5 reference material; we measured Fe as 4.197 ± 0.238 nmol L^-1^, within 95% confidence of the certified value (3.707 ± 0.627 nmol L^-1^).

#### Analysis of Variance

Analysis of variance (ANOVA) and least significant difference (LSD) were calculated using GenStat (Release Version 15.1) on each growth parameter for the three treatments for each species, testing whether the populations were significantly different from each other in each case.

## Results and Discussion

**Fig 1:**
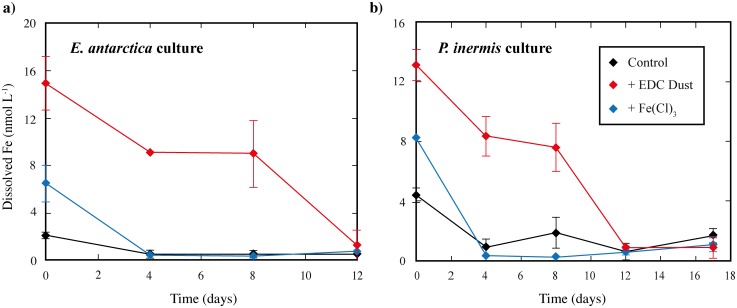
Dissolved Fe concentration (nmol L^-1^) measured in cultures of a) *Eucampia antarctica* or b) *Proboscia inermis* under different conditions (control, +EDC Dust, +FeCl_3_) by Flow Injection Analysis. The initial values were measured in the 8 L canister, prior to subdivision into five 1 L bottles, and these values are shown for time = 0. After this, the mean of measurements in the replicate bottles is shown (n = 3 or n = 5 for final measurement), with error bars denoting ± 1SD (where bigger than the size of the symbol).

**Fig 2:**
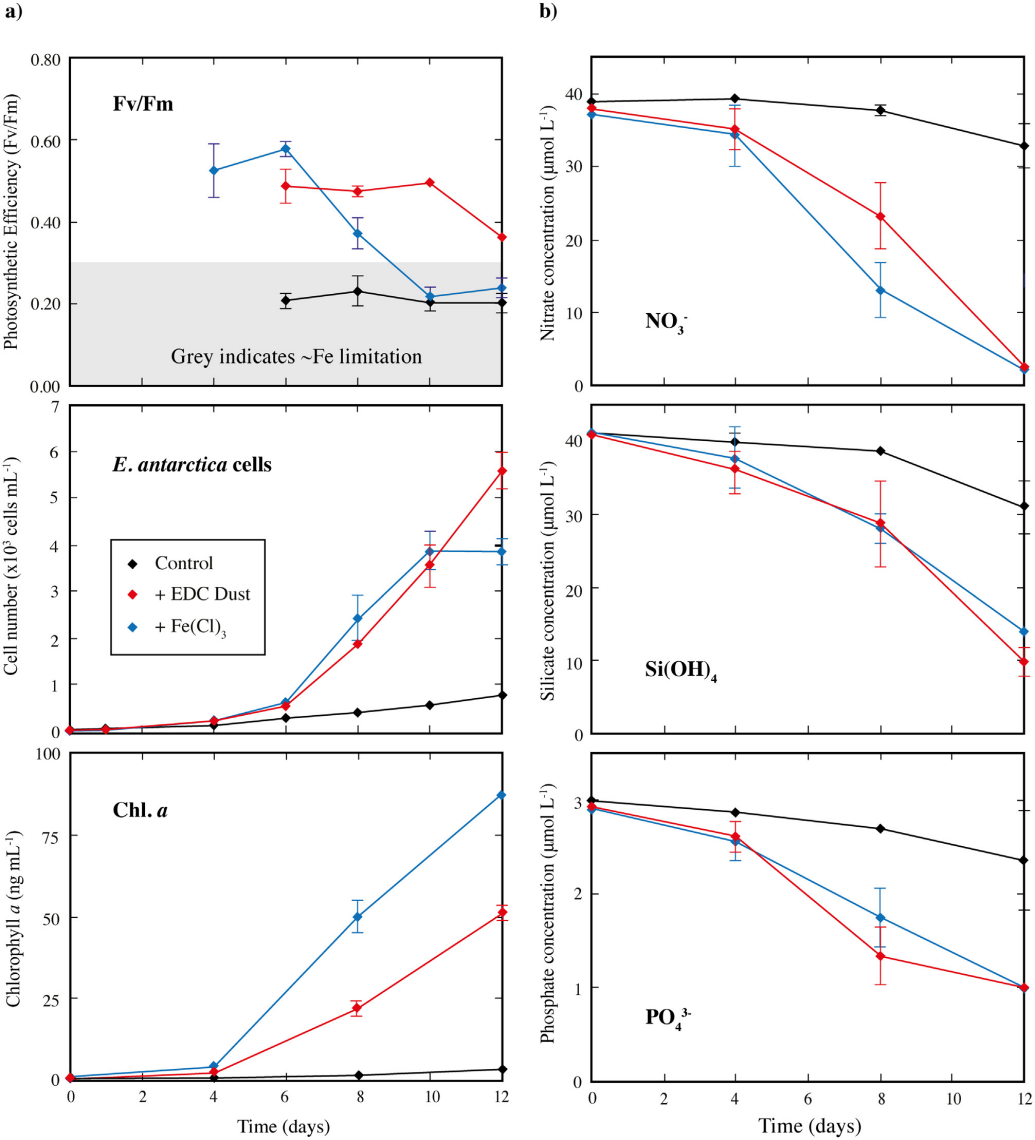
**a) The growth of *Eucampia antarctica* under different conditions (control or in response to iron chloride (+FeCl**_**3**_**) or dust (+dust) addition), over 12 days.** Photosynthetic efficiency (Fv/Fm), diatom cell number and chlorophyll *a* concentration are shown. **b) Nutrient concentration in different incubations of *E*. *antarctica* over the course of each experiment.** Nitrate (NO_3_^-^), silicate (Si(OH_4_)) and phosphate (PO_4_^3-^) are shown. The initial values were measured in the 8 L canister, prior to subdivision into five 1 L bottles, and these values are shown for time = 0. After this, the mean of measurements in the replicate bottles is shown (n = 3 or n = 5 for final measurement), with error bars denoting ± 1SD (where bigger than the size of the symbol). The grey bar indicates Fv/Fm values that are considered to represent cells under stress.

**Fig 3:**
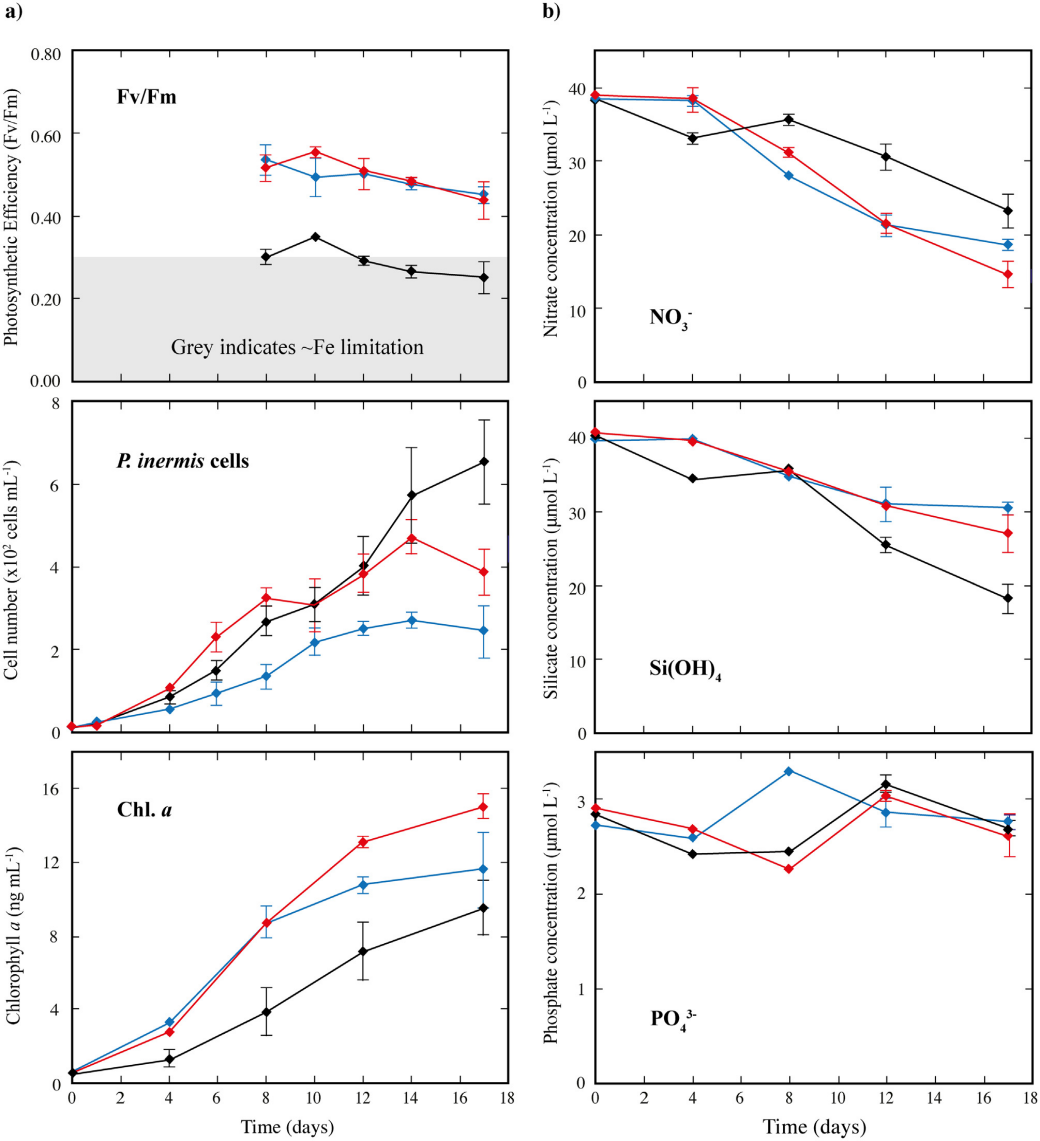
**a) The growth of *Proboscia inermis* under different conditions (control or in response to iron chloride (+FeCl**_**3**_**) or dust (+dust) addition), over 17 days.** Photosynthetic efficiency (Fv/Fm), diatom cell number and chlorophyll *a* concentration are shown. **b) Nutrient concentration in different incubations of *P*. *inermis* over the course of each experiment.** Nitrate (NO_3_^-^), silicate (Si(OH_4_)) and phosphate (PO_4_^3-^) are shown. For each parameter initial values were measured in the canister, before subdivision of the culture medium into 5 bottles, and these are shown for time = 0. After this, the mean of measurements in the replicate bottles is shown (n = 3 for 2–10days, n = 5 at 17 days), with error bars denoting ± 1SD (where bigger than the size of the symbol). The grey bar indicates Fv/Fm values that are considered to represent cells under stress.

**Fig 4:**
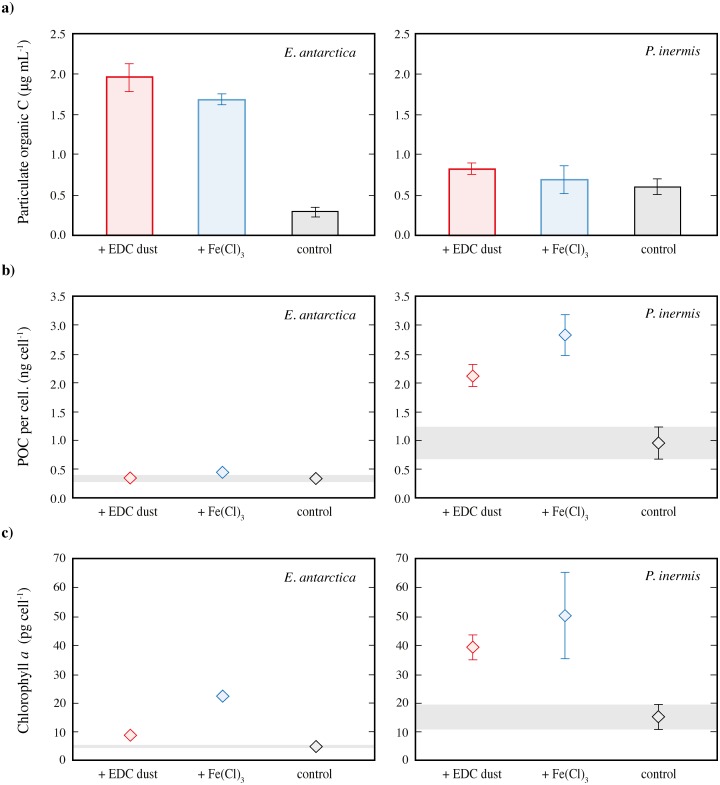
Organic carbon and Chlorophyll *a* produced by *Eucampia antarctica* and *Proboscia inermis* during incubations. **a)** final particulate organic carbon concentration in each experiment (POC; μg mL^-1^). **b)** final particulate organic carbon per diatom cell (POC; ng cell^-1^). **c)** final Chlorophyll *a* concentration in each incubation (μg mL^-1^). For each parameter, the mean of 5 replicate bottle measurements is shown, with error bars denoting ± 1SD (where bigger than the size of the symbol).

The mean results (with 1SD error bars) are shown in Figs 1–4 and Tables 1 and 2, with the full dataset available in S1 Data. Data within text is described using the mean values for each parameter ± 1 SD. Populations are described as different or similar based on testing with ANOVA, and the least significant difference typically at the 99% confidence limit.

**Table 1:** Measured *Eucampia antarctica* and *Proboscia inermis* growth parameters (cell number, chlorophyll *a*, particulate organic carbon [POC] and nutrient uptake) under different conditions (control, + EDC dust, +FeCl_3_). Mean values are shown, calculated from 5 replicate bottles, ±1 SD. Initial chlorophyll *a* concentration was calculated from a single dilution of initial cell cultures. Nutrient uptake was calculated from the reduction in nutrient concentration in solution from beginning to end of experiment. Statistical difference from the control was tested with Analysis of Variance (ANOVA; see Methods), and p values and Least Significant Difference (LSD) are shown.

Diatom Species	Condition	Final cell number	Initial chl. *a*	Final chl. *a*	Silicate uptake	Nitrate uptake	Phosphate uptake	Final POC
		*cells mL*^*-1*^	*μg L*^*-1*^	*μg L*^*-1*^	*μmol*	*μmol*	*μmol*	*μg mL*^*-1*^
***E*. *antarctica***	Control	786 ±76	0.31	3.90 ± 0.42	10.1	6.19	0.88	0.28 ± 0.05
	+ dust	5620 ± 408	0.31	51.3 ± 2.15	31.1	35.6	1.95	1.96 ± 0.17
	+FeCl_3_	3880 ± 290	0.28	87.1 ± 1.54	27.2	34.9	1.91	1.68 ± 0.07
	*ANOVA p value*	<0.001	-	<0.001	<0.001	<0.001	<0.001	<0.001
	*LSD*	403	-	2.13	3.67	2.75	0.38	0.15
***P*. *inermis***	Control	654 ± 104	0.56	9.57 ±1.53	22.3	15.1	0.16	0.60 ± 0.09
	+dust	388 ± 55	0.56	15.1 ± 0.68	13.7	24.4	0.31	0.82 ± 0.07
	+FeCl_3_	244 ± 62	0.71	11.6 ± 2.07	9.25	19.7	-	0.69 ± 0.17
	*ANOVA p value*	<0.001	-	<0.001	<0.001	<0.001	0.019	0.038
	*LSD*	106	-	2.11	3.00	1.24	0.19	0.16

**Table 2:** Final composition of *Eucampia antarctica* and *Proboscia inermis* cells, and calculated nutrient uptake ratios under different conditions (control, + EDC dust, +FeCl_3_). Mean values are shown, calculated from 5 replicate bottles, ±1 SD. Nutrient uptake ratios were calculated from the reduction in nutrient concentration in solution from beginning to end of experiment (see Table 1). Statistical difference from the control was tested with Analysis of Variance (ANOVA; see Methods), and p values and Least Significant Difference (LSD) are shown.

Diatom Species	Condition	Final Cell Composition				Nutrient Uptake Ratio			Nutrient/cell	
		chl. *a*/cell	POC/cell	Si:POC	Si:chl. *a*	Si:N	Si:P	N:P	Si	N
		*pg cell*^*-1*^	*ng cell*^*-1*^	*mol*:*mol*	*μmol*:*mg*	*mol*:*mol*	*mol*:*mol*	*mol*:*mol*	*nmol cell*^*-1*^	*nmol cell*^*-1*^
***E*. *antarctica***	Control	5.0 ± 0.6	0.36 ± 0.06	0.45	2.59	1.61	10.33	6.18	0.013	0.008
	+ dust	9.2 ± 0.5	0.35 ± 0.02	0.19	0.61	0.87	15.96	18.28	0.006	0.006
	+FeCl_3_	23 ± 1.8	0.44 ± 0.04	0.19	0.31	0.78	14.26	18.26	0.007	0.009
	*ANOVA p value*	*<0*.*001*	*0*.*036*	*0*.*008*	*<0*.*001*	*<0*.*001*	*<0*.*001*	*<0*.*001*	*0*.*008*	*0*.*357*
	*LSD*	*1*.*70*	*0*.*07*	*0*.*17*	*0*.*78*	*0*.*18*	*1*.*58*	*2*.*00*	*0*.*004*	*0*.*004*
***P*. *inermis***	Control	15 ± 4	0.95 ± 0.28	0.46	2.40	1.52	-	-	0.034	0.024
	+dust	39 ± 4	2.13 ± 0.19	0.20	0.91	0.56	-	-	0.036	0.064
	+FeCl_3_	50 ± 15	2.83 ± 0.36	0.17	0.82	0.47	-	-	0.039	0.084
	*ANOVA p value*	*<0*.*001*	*<0*.*001*	*<0*.*001*	*<0*.*001*	*<0*.*001*	-	-	*0*.*520*	*<0*.*001*
	*LSD*	*14*.*5*	*0*.*44*	*0*.*09*	*0*.*49*	*0*.*14*	-	-	*0*.*009*	*0*.*017*

### Dissolved Fe and Fv/Fm in incubations

#### Initial dissolved Fe

Dissolved Fe concentration (dFe) was measured in each canister before and after the addition of diatom cultures (Fig 1). In the control canisters, the mean [dFe] was 1.73 and 1.92 nmol L^-1^ prior to diatom addition, and 2.1 and 4.4 nmol L^-1^ for *E*. *antarctica* and *P*. *inermis* respectively after addition of culture (Fig 1). These initial control dFe concentrations are higher than would be expected in the open HNLC Southern Ocean (e.g. 0.05–0.10 nmol L^-1^; see introduction), but occurred despite our best efforts and deployment of trace metal clean procedures throughout. Typically culture studies use Fe-binding ligands such as EDTA or DFB to control free Fe and Fe-limit phytoplankton [37–39]. In this study, however, we chose not to use an Fe-binding ligand because the ligand might have enhanced dissolution of Fe from dust, or changed the speciation of Fe in solution and would have prevented us from measuring dFe. This decision meant that it was much more difficult to maintain cultures at open-ocean concentrations, given the necessary addition of nutrients and cultures. The increase from 0.2 to ~2 nmol L^-1^ could be the result of Fe contamination by handling or more-likely from the pre-cleaned nutrient solutions, whilst addition of *E*. *antarctica* culture contributed ~0.2 nmol L^-1^ and *P*. *inermis* contributed ~2 nmol L^-1^, despite these cultures being in an Fe-limited state prior to addition, based on Fv/Fm measurements.

However, despite the higher than expected initial dFe in the controls, dFe in the canisters following Fe/dust (and culture) addition was significantly higher, with 15 and 13 nmol L^-1^ for the +dust, and 6 and 8 for the +FeCl_3_ for *E*. *antarctica and P*. *inermis* respectively. The dFe concentrations added by dust addition were thus 9 and 11 nmol L^-1^ for *P*. *inermis* and *E*. *antarctica*, respectively. This compares reasonably well with the predicted increase in Fe addition based on [24]: the 0.02 mg Fe dust added should result in an average increase in dFe of ~32 nmol L^-1^ (range of 6 to 109 nmol L^-1^ based on soluble Fe range from [24]). Whilst the +FeCl_3_ treatments were designed to match the measured dFe in solution following just dust addition, the measured dFe in the +FeCl_3_ canisters were lower than these values. This was likely due to precipitation of the concentrated Fe(III) standard, an unavoidable consequence of diluting a concentrated acidic Fe(III) solution with higher pH ultrapure water and then seawater, where Fe(III) solubility is much lower and so ~50% of dissolved Fe precipitates rapidly (see Suppl. Disc. of [24], and [70]). This meant that the +FeCl_3_ incubations began with lower dFe than the +dust treatments (Fig 1). Nevertheless, the initial dFe concentrations in the +FeCl_3_ incubations were higher than the control incubations and still provided a useful comparison of Fe released from dust with Fe provided from a dissolved Fe^3+^ addition (Fig 1).

#### Dissolved Fe and Fv/Fm throughout the incubations

Despite high initial values in the control incubations, dFe concentrations for each species declined relatively quickly (Fig 1), reaching 0.51±0.30 and 0.91±0.58 nmol L^-1^ by day 4 in the control incubations for *E*. *antarctica* and *P*. *inermis* respectively. For *E*. *antarctica*, the control then remained at ~0.5 nmol L^-1^ until the end of the incubations, whilst for *P*. *inermis*, there was more variability, although dFe concentrations remained below 2 nmol L^-1^ (Fig 1). A similar pattern was observed in the +FeCl_3_ treatments, with dFe values of 0.3–0.8 nmol L^-1^ for *E*. *antarctica* and 0.3–1.1 nmol L^-1^ *P*. *inermis* from day 4 onwards (Fig 1). This reduction in measured dissolved Fe in both control and +FeCl_3_ treatments may indicate relatively rapid precipitation of dissolved Fe^3+^ species from solution, to near the organic ligand binding capacity of the seawater (0.5 nmol L^-1^), as well as uptake by diatom growth. Such a finding is consistent with experiments with seawater at ambient pH, which showed significant loss of naturally occurring or added dissolved Fe over the first 72–75 hours [71,70].

In contrast to the +FeCl_3_ and controls, the dFe concentrations in the +dust treatments remained high for the first 8 days of the experiments for both cultures (Fig 1). dFe concentrations decreased from 14.9 to 9 nmol L^-1^in the first 8 days in *E*. *antarctica* and from 13.9 to 9.5 nmol L^-1^ in *P*. *inermis*. Thereafter, dFe concentrations were similarly low in all treatments in both cultures (between 0.5 and 1.68 nmol L^-1^; Fig 1). Thus, whilst measured dFe did decline over the course of the experiment in the +dust treatments for both diatom species, dFe remained much higher than the controls over most of the sampling period. This may indicate that there was continuous inorganic or organic-mediated release of Fe from dust particles, or that the dissolved Fe released was in a form that was more easily stabilized or solubilized in seawater, such as Fe(II) species or Fe colloids which persist for longer [71]. The eventual decline to dFe values similar to control may then represent the uptake of Fe into diatom cells, or the conversion of soluble Fe species to those such as Fe hydroxides which may be more readily precipitated/adsorbed from solution [72,73].

Photosynthetic efficiencies (Fv/Fm) of <0.30 are generally taken to indicate stress or Fe-limitation of the diatom species [61,74]. By day 6 for *E*. *antarctica and day 8 for P*. *inermis*, the control incubations for both species showed significantly lower Fv/Fm values compared to the Fe addition treatments (Figs 2a and 3a). This difference was especially pronounced for *E*. *antarctica* (Fig 2a), with Fv/Fm values of just ~0.2 from days 6–12, and to a lesser extent for *P*. *inermis* (Fig 3a) with values of 0.2–0.4 from day 8–17. This observation, paired with the rapid reduction in dFe in the control incubations (Fig 1) to dFe concentrations where *E*. *antarctica* has been shown to be unable to grow (0.5 nmol L^-1^; [39]), suggests that the control incubations were Fe-limited by day 6–8, and at least for *E*. *antarctica*, perhaps from much earlier in the experiment (Fig 2a). By contrast, Fv/Fm remained high (>0.4) for *P*. *inermis* in both the +dust and +FeCl_3_ treatments over the course of the whole incubation period (Fig 3a), suggesting that neither culture was experiencing Fe limitation or stress. The same was true for *E*. *antarctica* in the +dust treatment over the course of the incubations, while for the +FeCl_3_ treatment Fv/Fm values declined to Fe-limited conditions similar to the control after day 10. Thus, the evidence suggests that, despite having initial dFe concentrations higher than open-ocean conditions, both diatom species experienced Fe-limitation or stress in the control incubations from early on the incubation experiments, a state that was alleviated in the +dust and at least initially in the +FeCl_3_ treatments.

#### Other Trace Metals

Although we did not measure the release of trace metals in the dust, it is possible they may have also affected growth, either acting as a toxin (e.g. Cd, Cu) or alleviating limitation (e.g. Mn, Zn) [75]. Although we can likely rule out toxicity from the other metals because Cd, Co, Cu, Zn and other metals are only found at low concentration (an order of magnitude less than Fe and Mn) from dust in the EDC core [76,77], and trace metal toxicity (e.g. Cu) is suppressed at relatively high levels of Mn^3+^ or Fe^3+^ [78], it is possible that Mn supplied from atmospheric dust could also contribute to a positive diatom growth response as recently hypothesized for volcanic ash [60].

### Physiological Response to Dust and Fe chloride addition

#### Eucampia Antarctica

In the control treatment of *E*. *antarctica*, cell numbers increased from 27 ± 3 cells mL^-1^ to 786 ± 76 mL^-1^ in 12 days and chlorophyll *a* concentrations increased from 0.31 to 3.9 ± 0.42 μg L^-1^ (Fig 2, Table 1). Also, nutrient drawdown was relatively low with 10.1, 6.19, and 0.88 μmol of silicate, nitrate, and phosphate taken up respectively (Fig 2, Table 1). By contrast, there was a strong growth response to dust addition in the +dust *E*. *antarctica* treatments, with Fv/Fm values typical of Fe replete conditions (0.4–0.5), a large increase in cell number from 27 ± 3 cells mL^-1^ to 5620 ± 408 mL^-1^ in 12 days and a chlorophyll *a* increase from 0.31 to 51.3 ± 2.15 μg L^-1^, coupled with rapid nutrient uptake of nitrate, phosphate and silicate of 31.1, 35.6, and 1.95 μmol, respectively (Fig 2, Table 1). Cell numbers and nutrient uptake were all significantly higher in the +dust treatments than the controls at the 99.9% confidence level (Table 1). Similarly, much more particulate organic carbon (POC) was produced in the +dust treatments compared to the controls (1.96 ± 0.17 compared to 0.28 ± 0.05 μg mL^-1^) (Figs 2 and 4; Table 1). These differences in chlorophyll *a* and POC concentration, which correspond to an increase of 13x and 6x, respectively in the +dust compared to the controls, are significantly different at the 99.9% confidence level.

A similar growth response as in the +dust treatments was observed for *E*. *antarctica* in the +FeCl_3_ treatments (Figs 1 and 3; Table 1). Here, we observed similarly high Fv/Fm values, a rapid nutrient drawdown of 27.2, 34.9 and 1.91 μmol silicate, nitrate and phosphate, respectively. Cell numbers increased from 27 ± 3 cells mL^-1^ to 3880 ± 290 mL^-1^ in 12 days, and POC concentrations from 0.01 to 1.68 ± 0.07 μg ml^-1^. This is a significant difference compared to the control for all mentioned parameters at the 99.9% confidence level (Table 1). While the nutrient uptake was very similar between the +dust and the +FeCl_3_ treatments, Fv/Fm, cell numbers and chlorophyll *a* concentrations showed some significant differences. We observed a greater final chlorophyll *a* concentration in the +FeCl_3_ treatments compared to the +dust (87.1 μg L^-1^ vs 51.3) although this was coupled with lower final Fv/Fm, cell counts and POC than in the +dust (ANOVA suggests significant at the 99.9% confidence level; Figs 1 and 3; Table 1).

In contrast to the +dust treatments, the strong growth in the +FeCl_3_ treatments did not persist for the full 12 days, with no increase in cell number after day 10 (Fig 2). Fv/Fm values declined from day 6 onwards, reaching values which are indicative of Fe-limitation (<0.3) by day 10 (Fig 2), likely reflecting the decline in dFe in the +FeCl_3_ treatments to those of the controls after day 4. Despite this decline in both Fv/Fm and dFe, *E*. *antarctica* cell counts in the +dust and +FeCl_3_ treatments were equivalent until day 10 (Fig 2). After day 10, however, the cell numbers in the +dust treatment continued to increase (with Fv/Fm >0.3) while in the +FeCl_3_ treatments there was no increase in cell number (Fig 2). Overall, the lower final cell counts in the +FeCl_3_ compared to the +dust treatments (3880 compared to 5620) corresponds to 1 less doubling of cells in the +FeCl_3_ treatment and is significantly different at the 99.9% confidence level (Table 1). Since Fe is directly required for production of the photosystems, chlorophyll *a* production is a good indicator that sufficient ‘bioavailable’ Fe is present. In this context it is interesting that significantly more chlorophyll *a* production was observed in the +FeCl_3_ treatment which had not only lower final cell numbers, but also lower dFe concentrations throughout the incubations (Fig 1a). This observation suggests that not all the Fe released from the dust in the operationally defined dFe parameter was bioavailable to *E*. *antarctica*, consistent with previous work on crystalline desert dust that documented that only a fraction of Fe released from dust may be bioavailable [79].

The low Fv/Fm values after day 10 in the +FeCl_3_ treatments, despite the fact that major nutrients were still present (Fig 2), suggests that diatoms in the +FeCl_3_ treatments began to experience Fe limitation by day 10, resulting in no further growth. It is, however, interesting to note that the decline in Fv/Fm lagged the onset of low dFe by several days, perhaps suggestive of ‘luxury uptake’ of Fe by diatoms during periods of higher Fe concentrations [80]. These high internal Fe concentrations can be passed on as cells divided and sustain growth for several days beyond the onset of Fe limitation. It is also interesting to note that the Fe-limitation inferred for the +FeCl_3_ and control treatments in the later stages of the incubations, based on Fv/Fm, corresponds to dFe concentrations of ~0.5 nmol L^-1^. This observation may suggest that the Fe requirements for *E*. *antarctica* are relatively high, or that not all the dFe was in a form available for utilization by the diatoms.

#### Proboscia inermis

For *P*. *inermis*, although low Fv/Fm values (~0.3) indicated Fe limitation in the control incubations by day 8, compared to higher values in the +dust and +FeCl_3_ treatments throughout the experiment (>0.4), there was no obvious response to dust or Fe addition in terms of cell number (Fig 3; Table 1); in fact, there were less cells in both the +FeCl_3_ and +dust treatments (388 ± 55 and 244 ± 62 cells mL^-1^) at the end of the experiment compared to the controls (654 ± 104 cells mL^-1^). Until day 14, there was almost no difference in the cell numbers between the control and the + dust treatment while cell numbers were lowest in the +FeCl_3_ treatment throughout (Fig 3). The similar growth of *P*. *inermis* both with and without dust or Fe is comparable to that observed previously by de Baar and Boyd [55], who documented no obvious growth response to +2 nmol L^-1^ dFe treatments for this species, although this is different to experiments by Strzepek *et al*. who found that *P*. *inermis* cells placed under Fe-limitation using the siderophore DFB to complex dissolved Fe (a ratio of 4:400 or 4:200 Fe:DFB) showed growth rates reduced by ~50%, or using seawater with 0.49 nmol L^-1^ showing a 67% reduction in growth rates [39,81]. Thus the lack of obvious growth response in our experiment in terms of cell number could indicate that *P*. *inermis* has a lower Fe requirement than the dissolved Fe present in any of our treatments, which might not be surprising given the relatively high dFe concentrations maintained in *P*. *inermis* cultures compared to *E*. *antarctica*. However, this would be in apparent disagreement with Fv/Fm measurements which suggest that the *P*. *inermis* control was ‘Fe-limited’, whilst the +dust and +FeCl_3_ treatments were not.

This apparent discrepancy could be resolved by investigation of other parameters, such as POC and chlorophyll *a* production. Indeed, in contrast to cell number results, final chlorophyll *a* concentration was higher in the +dust *P*. *inermis* treatments compared to the other *P*. *inermis* treatments. Both the +dust and the +FeCl_3_ treatments showed significantly higher chlorophyll *a* concentrations compared to the control but were not significantly different from each other until day 8. Thereafter, chlorophyll *a* concentrations were higher in the +dust treatment (Fig 3; Table 1). The mean POC concentration in the +dust treatments was also slightly higher (Table 1; Fig 4), but this is only significant at the 97.2% confidence level. Taken together, these results suggest that *P*. *inermis* exhibits a different response to Fe addition than *E*. *antarctica*, possibly as a result of luxury Fe uptake, as discussed in more detail below, or possibly because *E*. *antarctica* has a higher requirement for Fe than *P*. *inermis*, consistent with previous work which showed *E*. *antarctica* to be unable to grow at Fe:DFB ratios >4:50 whilst P. inermis could grow at ratios up to 4:200 [39]. However, we note that the growth of *P*. *inermis* in that study could also be because *P*. *inermis* is better able to acquire Fe from DFB by Fe-reduction [39] than *E*. *antarctica*.

Notably, in contrast to *E*. *antarctica*, *P*. *inermis* diatoms in the +FeCl_3_ treatment produced overall less chlorophyll *a* than those with the +dust, perhaps suggesting that Fe from dust was more ‘bioavailable’ to *P*. *inermis* than *E*. *antarctica* (or that the dissolved Fe precipitated more rapidly in *P*. *inermis* treatments). However, in considering the implications of this result for bioavailability of Fe from dust for *P*. *inermis*, we must also consider the overall number of diatoms cells which were significantly fewer in the +FeCl_3_ treatment (see below).

### Diatom Cell Composition

#### Chlorophyll *a* or particulate organic carbon per cell

**Fig 5:**
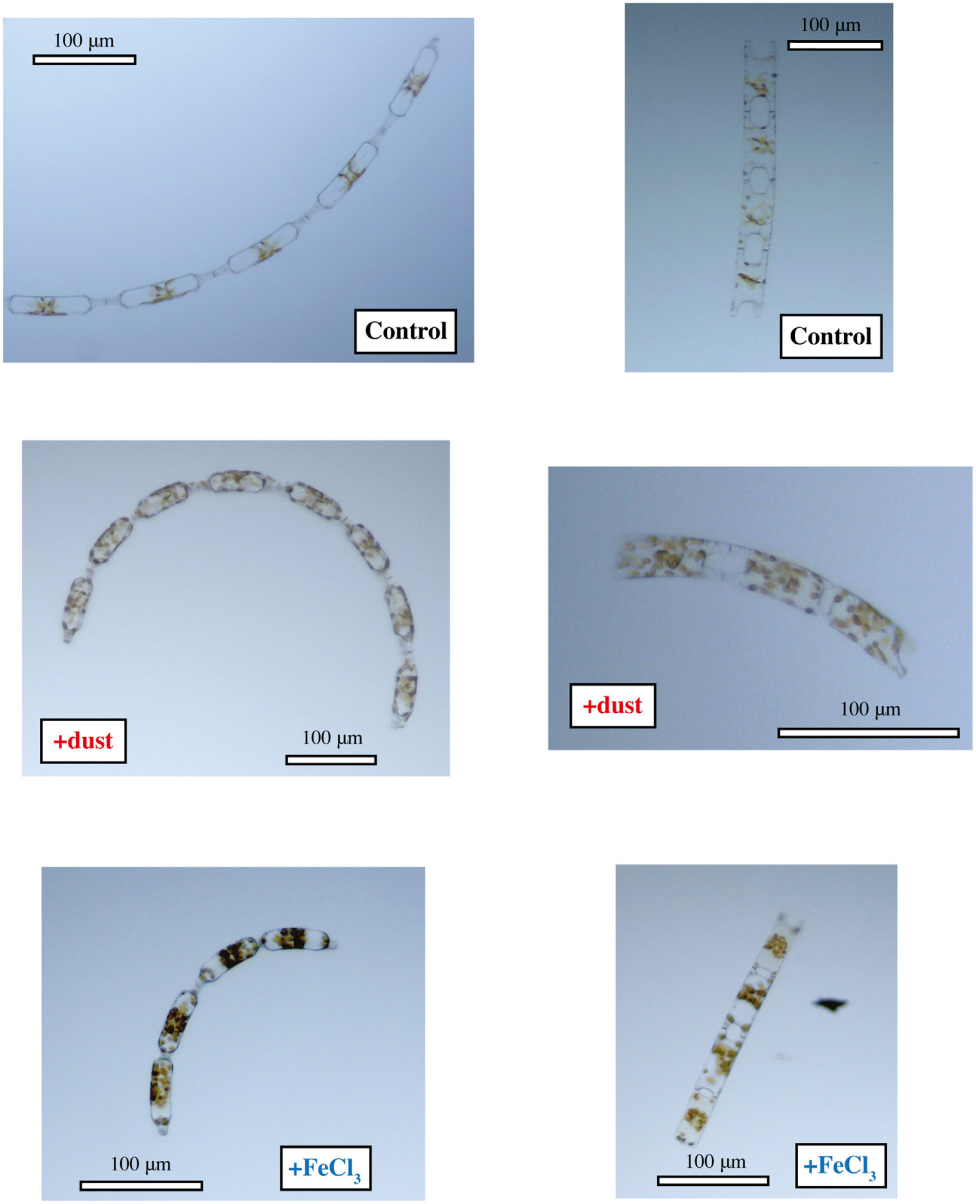
Photomicrographs of *Eucampia antarctica* cells, showing higher number and density of chloroplasts in amended treatments. Panels are labeled control, +dust or +FeCl_3_ to denote the incubation conditions.

**Fig 6:**
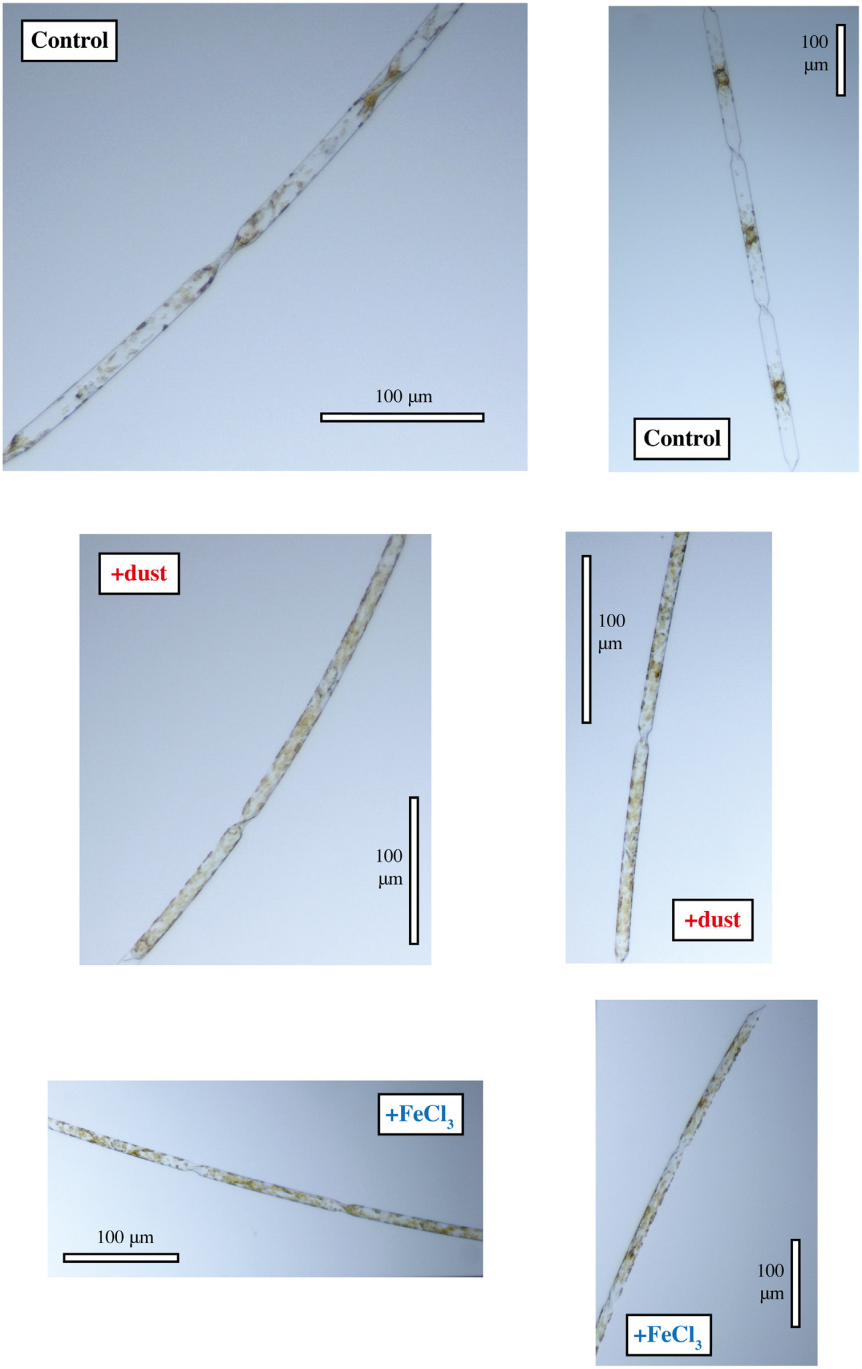
Photomicrographs of *Proboscia inermis* cells, showing higher number and density of chloroplasts in amended treatments. Panels are labeled control, +dust or +FeCl_3_ to denote the incubation conditions.

Cellular carbon and chlorophyll *a* concentrations and nutrient uptake for the final day of the incubations are shown in Table 2, Fig 4 and cell volume is shown in S1 Data. For *E*. *antarctica*, the concentrations of chlorophyll *a* per cell in the +dust treatment were about twice as high as those in the control and more than four times higher than the control in the +FeCl_3_ treatment (Fig 4c). For *P*. *inermis*, chlorophyll *a* concentrations per cell in both +dust and +FeCl_3_ treatments were statistically equivalent and more than two times higher compared to the control (Fig 4c, Table 2). This shows that both diatom species increased chlorophyll *a* production dramatically in response to either FeCl_3_ or dust addition, something that was not obvious from total chlorophyll *a* production alone in the case of *P*. *inermis*. This increase in chlorophyll *a* production can also be further observed in photomicrographs showing an increased number of chloroplasts per cell in the +FeCl_3_ and +dust treatments for both species compared to the controls (Figs 5 and 6).

The POC production by *P*. *inermis* was only slightly lower in the control compared to the other two treatments, but the difference was not significant (Fig 4a, Table 1). However, because of the lower cell numbers in the +FeCl_3_ and +dust treatments, the POC concentration per cell was significantly higher in the +dust and +FeCl_3_ treatments by more than a factor of two (Table 2, Fig 4b), and highest in the +FeCl_3_ treatments. This shows that POC fixation increased for *P*. *inermis* in response to Fe addition. Conversely, in *E*. *antarctica* overall POC concentrations at the end of the experiment were six to seven times higher in response to dust or FeCl_3_ addition, respectively compared to the control (Table 1, Fig 4a). The equivalent POC concentrations per cell, however, showed no significant difference between the three treatments. These observations highlight the differing response of the two species to Fe addition, with *E*. *antarctica* showing a strong positive growth response, producing more cells and more chlorophyll *a* but not increasing relative carbon uptake per cell. In contrast, *P*. *inermis* has a more subtle response, fixing more carbon and chlorophyll *a* per cell but perhaps not increasing cell number. However, lower Fe concentration experiments might be required to test this definitively. Furthermore, the composition of the cells of both species does suggest a stronger growth response to FeCl_3_ than dust addition, supporting the previous assertion that not all Fe released from dust was bioavailable.

#### Nutrient uptake ratios

Both species demonstrated a statistically different change in their nutrient uptake ratios in response to FeCl_3_ or dust addition, compared to the controls (Table 2). For *E*. *antarctica*, the Si:N ratio dropped from 1.61 in the control to 0.87 for +dust and 0.78 for +FeCl_3_ (with these identical at the 99.9% confidence level). Similarly, *P*. *inermis* cells changed their Si:N uptake ratio from 1.52 in the control to 0.56 and 0.47 in the +dust and +FeCl_3_ treatments (again statistically identical). However, the reason for these nutrient uptake changes differs between the two species. For *E*. *antarctica* the silicate/cell decreased by ~2x for the +FeCl_3_ or +dust treatments relative to cells in the control incubations, but nitrate/cell showed no significant difference between the three treatments (Table 2). By contrast, for *P*. *inermis*, the silicate/cell was the same for all three treatments, but the +FeCl_3_ and +dust incubations showed marked increases in nitrate/cell (3-4x) relative to cells in the control incubations (Table 2).

A similar pattern is observed for Si:POC or Si:chlorophyll *a*, with both species dramatically reducing ratios in response to +Fe or +dust but again for opposing reasons (Table 2); *E*. *antarctica* reduced silicate/cell relative to POC/cell or chlorophyll *a*/cell in response to Fe or dust, while in contrast *P*. *inermis* increased POC/cell and chlorophyll *a*/cell for the same uptake of silicate/cell. This means that although both species increased their carbon fixation relative to silicate in response to both FeCl_3_ or dust, *E*. *antarctica* produced lighter less-silicate rich cells while *P*. *inermis* fixed more carbon and nitrate for the same amount of silicate. So while these changes in macronutrient uptake ratios are consistent with observation in both open ocean or under laboratory conditions, where diatoms grown under Fe replete conditions uptake relatively less Si compared to C and N, resulting in relatively less silicified diatom frustules [82–87], it is interesting that the reason for this varies. However, it is perhaps in line with the response of the two species, with *E*. *antarctica* producing more cells in response to Fe or dust, while *P*. *inermis* instead changed cell composition. Despite this difference in response, the fact that a change in Si:N ratio was observed for *P*. *inermis* in this experiment thus also lends support for the assertion that the control incubations were at least partially Fe-limited.

*E*. *antarctica* also significantly changed Si:P (14–16) and N:P (18) ratios in response to both Fe and dust, compared to the controls (10 and 6 for Si:P and N:P respectively; Table 2). Negligible uptake of phosphate in the *P*. *inermis* cultures meant that it was not possible to tell how phosphate uptake varied with Fe addition in these experiments.

## Conclusions and Implications

We have presented incubation experiments of the diatoms *E*. *antarctica* and *P*. *inermis* with glacial-age atmospheric dust extracted from the EDC ice core. Both diatom species demonstrated a different response to dust or FeCl_3_ addition. *E*. *antarctica* increased cell number, total chlorophyll *a* and total POC production when given Fe or dust, but maintained similar chlorophyll *a*:cell and POC:cell ratios. By contrast, *P*. *inermis* strongly increased chlorophyll *a*:cell and POC:cell but did not increase the overall number of cells in response to dust or Fe addition. The different responses of two diatom species is consistent with previous studies on the role of Fe and crustal desert dust addition on several diatom species [38,79], highlighting the fact that different species would respond differently to an enhanced atmospheric dust supply, with implications for changes in overall community structure. As phytoplankton community composition is known to have a major influence on carbon export [88], it is important to better understand the different ecological strategies of individual phytoplankton species in response to Fe fertilization. Nevertheless, the net result by both species in response to both Fe or dust was to produce more POC and chlorophyll *a*, together with the production of frustules with lower Si:C and Si:N ratios. Thus, these results confirm that the nutrient-utilization changes due to Fe addition which have been previously documented for diatoms with dissolved Fe changes in modern HNLC regions [87], would also have occurred due to enhanced deposition of atmospheric dust at the LGM. This observation also lends weight to hypotheses that suggest relief of Fe-limitation during glacial intervals could change the organic carbon:silicate ratio of diatoms [89]. This will likely be different between species and as such will have implications for weight of frustules and organic C:Si being exported from the surface ocean, the residence time of frustules in sediments and the use of opal records as paleoproxy indicators of surface productivity [89]. Furthermore, the lower silicate utilization relative to carbon in the Southern Ocean could have resulted in silicate being transported to and utilized at lower latitudes of the surface oceans with a consequent reduction in atmospheric CO_2_ levels [90,89,91].

## Supporting Information

S1 Data Supporting Data. (XLSX)

S1 Fig Apparatus for sublimation of ice-core material for dry extraction of large masses of ice core dust (based on [24]). (TIF)

S1 Text Calculating *Eucampia antarctica* cell volume. (DOCX)
