# Supplementary figures and images for: The Growth Response of Two Diatom Species to Atmospheric Dust from the Last Glacial Maximum
Source: PLoS One. 2016 Jul 6;11(7):e0158553. doi: 10.1371/journal.pone.0158553 (PMC4934930; doi:10.1371/journal.pone.0158553)

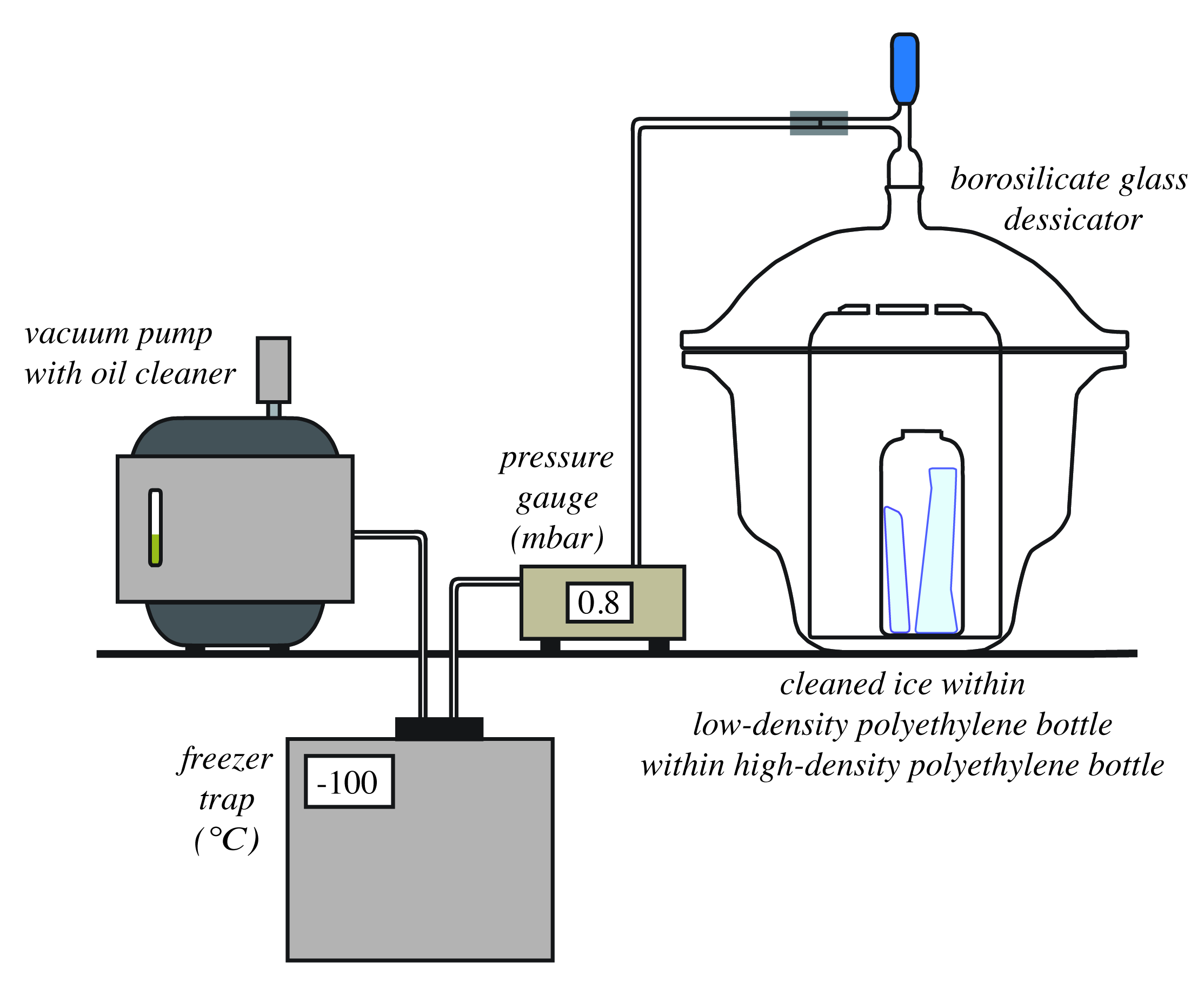

Supplement: S1 Fig — (TIF) [file pone.0158553.s002.tif]
