# Supplementary material for: The Growth Response of Two Diatom Species to Atmospheric Dust from the Last Glacial Maximum
Source: PLoS One. 2016 Jul 6;11(7):e0158553. doi: 10.1371/journal.pone.0158553 (PMC4934930; doi:10.1371/journal.pone.0158553)
Supplement: S1 Text — (DOCX) [file pone.0158553.s003.docx]

**Text S1. Calculating *Eucampia antarctica* cell volume**

In order to calculate volume for *Eucampia antarctica* cells, we assumed that cells could be approximated as a combination of simple ideal shapes. Because of the more-complex shape of centric diatom cells, we modeled the centric diatom *E. antarctica* as a combination of an ideal elliptical prism and four cylinders (see Fig. S2).


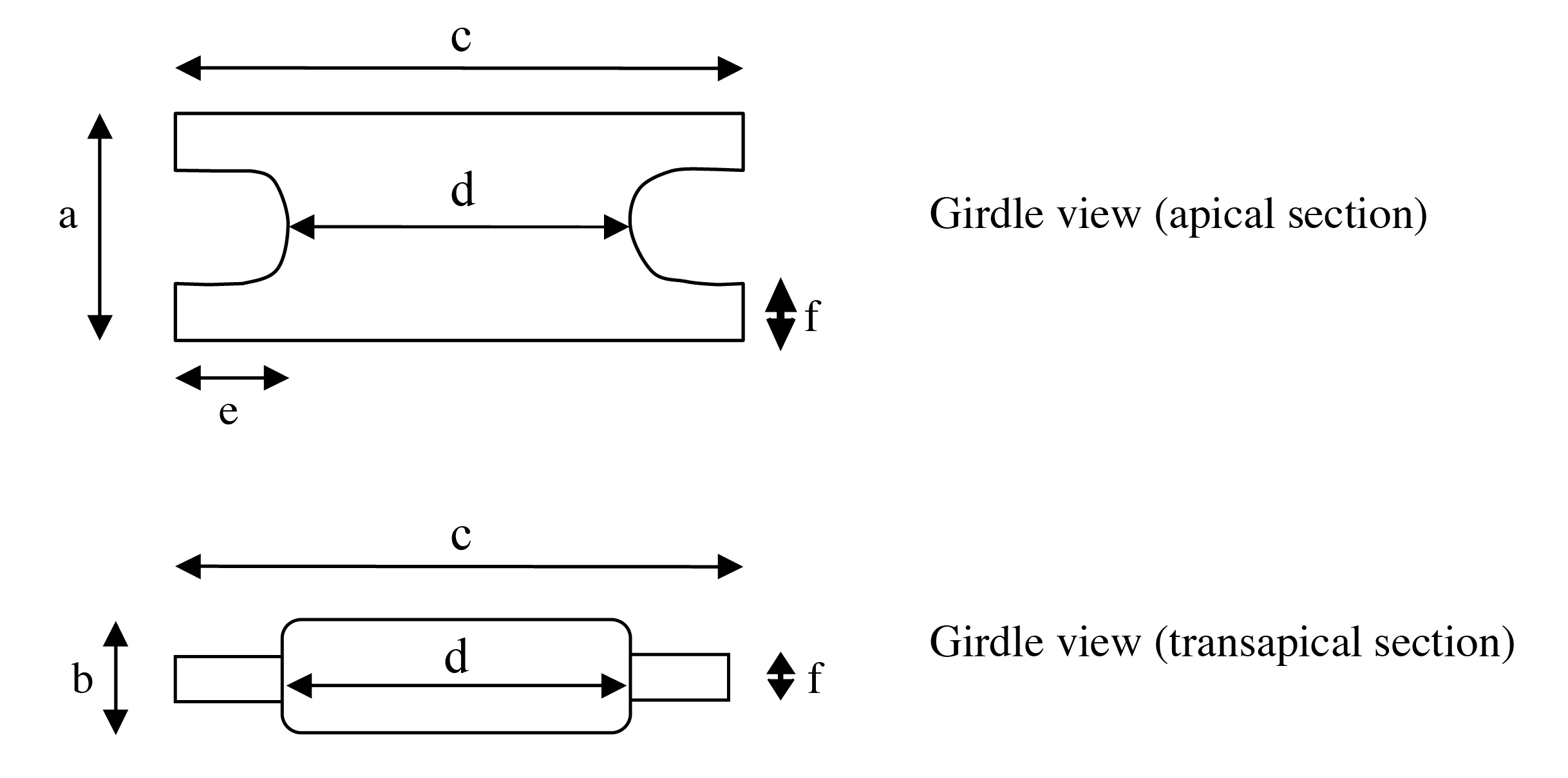


**Figure S2. Idealised schematic of an *Eucampia antarctica* cell.**

**Cell volume**

The volume of *E. antarctica* cells was calculated using eqn. 2, with dimensions shown in S. Fig. 1:

$$Cell volume= \frac{\pi}{4}\left( abd \right)+4\left( \frac{\pi}{4}f^{2}e \right) [2]$$
